# Excess body weight increases the burden of age-associated chronic diseases and their associated health care expenditures

**DOI:** 10.18632/aging.100833

**Published:** 2015-10-29

**Authors:** Vincenzo Atella, Joanna Kopinska, Gerardo Medea, Federico Belotti, Valeria Tosti, Andrea Piano Mortari, Claudio Cricelli, Luigi Fontana

**Affiliations:** ^1^ Department of Economics and Finance, University of Rome "Tor Vergata”, Rome, Italy; ^2^ Center for Health Policy, Stanford University, Stanford, CA 94305, USA; ^3^ Italian College of General Practitioners (SIMG), Florence, Italy; ^4^ Department of Medicine, Washington University School of Medicine, St. Louis, MO 63110, USA; ^5^ Department of Clinical and Experimental Sciences, Brescia University, Italy; ^6^ CEINGE Biotecnologie Avanzate, Napoli, Italy

**Keywords:** cost analysis, body mass index, disease burden, cardiovascular disease, diabetes, hypertension, obesity

## Abstract

Aging and excessive adiposity are both associated with an increased risk of developing multiple chronic diseases, which drive ever increasing health costs. The main aim of this study was to determine the net (non‐estimated) health costs of excessive adiposity and associated age‐related chronic diseases. We used a prevalence‐based approach that combines accurate data from the Health Search CSD‐LPD, an observational dataset with patient records collected by Italian general practitioners and up‐to‐date health care expenditures data from the SiSSI Project. In this very large study, 557,145 men and women older than 18 years were observed at different points in time between 2004 and 2010. The proportion of younger and older adults reporting no chronic disease decreased with increasing BMI. After adjustment for age, sex, geographic residence, and GPs heterogeneity, a strong J‐shaped association was found between BMI and total health care costs, more pronounced in middle‐aged and older adults. Relative to normal weight, in the 45‐64 age group, the per‐capita total cost was 10% higher in overweight individuals, and 27 to 68% greater in patients with obesity and very severe obesity, respectively. The association between BMI and diabetes, hypertension and cardiovascular disease largely explained these elevated costs.

## INTRODUCTION

The current obesity epidemic in an increasingly aging population presents health, long-term care, and welfare systems with new challenges [1]. Increased consumption of energy-dense, nutrient-poor foods and a sedentary lifestyle have led to this sharp and unprecedented rise in the rates of overweight and obesity, which has been estimated to increase both direct and indirect health care costs due to lost productivity [2, 3]. Aging and excess adiposity are both well-established risk factors for the development and progression of several chronic diseases, including type 2 diabetes, hypertension, dyslipidaemia, cardiovascular disease (CVD), osteoarthritis, depression and certain prevalent cancers (i.e. colon, breast, and prostate) [4]. The diagnosis and treatment of these common and preventable chronic diseases places a significant burden on National Health Service budgets. However, little is known about the true (non-estimated) impact of body mass index (BMI) on the inpatient and outpatient health care costs for these adiposity-associated chronic diseases. This study has several advantages over most existing studies. The majority of studies published so far have estimated the total health care costs of obesity by modelling group and individual level data with various degree of representativeness at national level, often relying on self-reported clinical information and proxies of medical expenditures [5-11]. In this study, we used a patient-based approach combining health care cost data and accurate anthropometric and clinical informations collected by general practitioners in a large representative longitudinal sample of more than 550 thousand Italian men and women, homogeneously distributed across all Italian regions. Moreover, Italy is an ideal setting for this type of analysis, since its National Health Service provides universal and substantially free health care access to all citizens, with 87% of medical services publicly financed [12], avoiding problems of patient selection associated with insurance based health care systems. Finally, by modelling this data with a seemingly unrelated regression equation (SURE) statistical method, we were able to disentangle the direct and indirect (i.e. obesity-associated diseases) impact of BMI on health care system spending.

## RESULTS

### Sample descriptive statistics

Table 1 summarizes the breakdown of the variables according to BMI classes, showing large differences in demographic and clinical parameters such as age and prevalence of comorbidities. Hypertension and type 2 diabetes were the most common BMI-associated health conditions and their prevalence shows a strong increase with increasing BMI (p=0.0001 for all BMI categories with respect to normal weight individuals). In addition, the prevalence of dyslipidaemia, CVD and arthrosis were higher in individuals with overweight and obesity than in normal weight individuals in both younger (<55 yrs) and older (>55 yrs) patients (p=0.0001 for all BMI categories with respect to normal weight individuals). There was a clear negative association between BMI and the proportion of individuals with no chronic disease (p=0.0001 for all BMI categories with respect to normal weight individuals). In contrast, the proportion of individuals affected by 2 or more chronic diseases increased sharply with raising BMI.

**Table 1:** Demographic and clinical characteristics of individuals 18 years or older, by BMI categories

	Under weight	Normal weight	Over Weight	Obesityclass I	Obesityclass II	Obesity class III
**BMI**	(15.00-18.49)	(18.50-24.99)	(25.00-29.99)	(30.00-34.99)	(35.00-39.99)	(≥ 40.00)
						
**Sample size**	72032	1103594	997802	390109	105731	35943
**Mean BMI (kg/m^2^)**	17.5*	22.3	27.2*	32*	36.9*	43.6*
**Gender (% female)**	82.3%*	61.0%	44.8%*	50.2%*	62.4%*	71.7%*
**Mean age (yrs)**	39*	48	56*	57*	56*	54*
**Age group (%)**						
**18-24**	25%*	10%	3%*	2%*	2%*	3%*
**25-34**	28%*	17%	8%*	7%*	7%*	8%*
**35-44**	19%*	20%	15%*	14%*	14%*	15%*
**45-54**	9%*	17%	19%*	19%*	20%*	21%*
**55-64**	6%*	13%	21%*	24%*	25%*	26%*
**65-74**	6%*	11%	19%*	21%*	21%*	19%*
**75+**	8%*	10%	14%*	14%*	12%*	8%*
**Comorbidities (%)**						
**Age group under 55**						
**Diabetes**	0.4%*	1.1%	3.5%*	7.3%*	12.1%*	16.7%*
**Hypertension**	1.9%*	6.4%	17.4%*	27.8%*	36.5%*	42.5%*
**Dyslipidemia**	1.9%*	5.4%	11.6%*	13.3%*	12.0%*	9.4%*
**CVD**	0.2%*	0.6%	1.5%*	2.0%*	2.2%*	2.0%*
**Depression**	4.7%*	4.0%	3.9%*	4.6%*	5.6%*	6.7%*
**Cancers**	0.5%*	0.6%	0.6%	0.7%	0.7%	0.7%
**Arthrosis**	0.9%*	1.2%	2.0%*	2.7%*	3.7%*	4.9%*
**Age group over 55**						
**Diabetes**	5.6%*	12.5%	19.9%*	28.0%*	35.0%*	38.7%*
**Hypertension**	38.9%*	49.4%	61.9%*	72.0%*	78.9%*	82.8%*
**Dyslipidemia**	18.8%*	26.9%	30.3%*	29.8%*	28.0%*	23.1%*
**CVD**	9.9%*	11.4%	13.9%*	14.7%*	14.6%*	12.3%*
**Depression**	10.4%*	8.0%	6.7%*	7.2%*	8.1%	8.3%
**Cancer**	6.5%*	6.0%	5.7%*	5.3%*	4.7%*	4.1%*
**Arthrosis**	6.5%*	8.0%	10.2%*	13.1%*	15.9%*	18.0%*
**No. of comorbidities**						
**Age group under 55**						
**0**	90.5%*	83.5%	68.5%*	58.1%*	50.3%*	45.4%*
**1**	8.7%*	14.0%	24.0%*	29.0%*	31.8%*	33.3%*
**2**	0.8%*	2.2%	6.1%*	9.9%*	13.4%*	15.5%*
**>=3**	0.1%*	0.3%	1.4%*	3.1%*	4.4%*	5.8%*
**Age group over 55**						
**0**	37.6%*	28.0%	19.3%*	13.4%*	9.9%*	8.5%*
**1**	37.0%	36.6%	35.4%*	33.1%*	31.0%*	31.1%*
**2**	18.4%*	23.6%	28.0%*	30.8%*	32.3%*	33.8%*
**>=3**	7.1%*	11.8%	17.4%*	22.8%*	26.8%*	26.6%*
**Smokers (%)**	44%*	40%	34%*	31%*	31%*	28%*
**(subsample in which smoking information was available)**	8124	138211	135669	54825	15007	5013

* p<0.01

† p<0.05

‡ p<0.1, each *p value* refers to a t test for equality of means for each category with respect to normal weight category

### BMI and healthcare costs

Table 2 and Table 3 present the coefficient estimates of indirect (i.e. obesity-associated diseases), direct and differential effects of BMI on outpatient and total health care costs for each age group. The tables report marginal effects for each BMI category within each age specific subsample, as well as the percentage differences of the marginal effect estimate with respect to the annual average expenditure of normo-weight individuals.

**Table 2:** Effects of BMI on “Outpatient” health expenditure with respect to normo‐weight individuals

	Indirect		Direct		Overall	
	Marginal effect	% difference from the mean	Marginal effect	% difference from the mean	Marginal effect	% difference from the mean
**Full sample (N=2705211), average estimated expenditure = 445 euro**						
**Underweight**	−8.918***	−2%	12.66***	3%	3.745*	1%
**Overweight**	46.19***	10%	−1.650**	0%	44.54***	10%
**Obesity**	98.07***	22%	12.51***	3%	110.6***	25%
**Severe obesity**	139.3***	31%	33.77***	8%	173.0***	39%
**Very severe obesity**	159.3***	36%	69.48***	16%	228.7***	51%
**18-44 (N=934147), average estimated expenditure = 173 euro**						
**Underweight**	−6.019***	−3%	−8.615***	−5%	−14.63***	−8%
**Overweight**	18.08***	10%	10.40***	6%	28.48***	17%
**Obesity**	39.05***	23%	18.44***	11%	57.48***	33%
**Severe obesity**	63.34***	37%	30.43***	18%	93.76***	54%
**Very severe obesity**	91.40***	53%	73.45***	43%	164.8***	95%
**45-64 (N=990831), average estimated expenditure = 416 euro**						
**Underweight**	−33.64***	−8%	30.61***	7%	−3.034	−1%
**Overweight**	74.71***	18%	1.289	0%	76.00***	18%
**Obesity**	145.1***	35%	14.16***	3%	159.3***	38%
**Severe obesity**	203.9***	49%	33.27***	8%	237.2***	57%
**Very severe obesity**	236.3***	57%	73.93***	18%	310.2***	75%
**65+ (N=780233), average estimated expenditure = 796 euro**						
**Underweight**	−84.66***	−11%	−0.941	0%	−85.60***	−11%
**Overweight**	65.49***	8%	5.145***	1%	70.64***	9%
**Obesity**	115.8***	15%	25.25***	3%	141.1***	18%
**Severely obesity**	145.9***	18%	56.22***	7%	202.1***	25%
**Very severe obesity**	138.3***	17%	78.01***	10%	216.3***	27%

*** p<0.01

** p<0.05

* p<0.1, each *p value* refers to a t test for equality of means for each category with respect to normal weight category.

Average estimated expenditure for each sample was obtained as the predicted expenditure from the health expenditure equation, calculated for the normal weight individuals, and at means of all other regressors.

Indirect marginal effects for each BMI category were computed as the sum of nonlinear combinations of parameters estimated within each pathology-specific equation with the respective pathology-specific parameter estimated within the health expenditure equation.

Direct marginal effects for each BMI category were obtained as relative parameter estimates from the health expenditure equation.

verall marginal effects for each BMI category was computed as the sum of the respective direct and indirect marginal effects.

**Table 3:** Effects of BMI on “Total Outpatient and Inpatient” health expenditure with respect to normo-weight individuals

	Indirect		Direct		Overall	
	Marginal effect	% difference from the mean	Marginal effect	% difference from the mean	Marginal effect	% difference from the mean
**Full sample (N=2705211), average estimated expenditure = 1092 euro**						
**Underweight**	−15.92***	−1%	154.0***	14%	138.1***	13%
**Overweight**	104.0***	10%	−66.52***	−6%	37.44***	3%
**Obesity**	237.3***	22%	−43.89***	−4%	193.4***	18%
**Severe obesity**	351.2***	32%	95.02***	9%	446.2***	41%
**Very severe obesity**	417.1***	38%	133.0***	12%	550.1***	50%
**18-44 (N=934147), average estimated expenditure = 399 euro**						
**Underweight**	−10.23***	−3%	72.71***	18%	62.49***	16%
**Overweight**	30.42***	8%	−8.392***	−2%	22.03***	6%
**Obesity**	66.72***	17%	5.606**	1%	72.32***	18%
**Severe obesity**	108.8***	27%	47.43***	12%	156.3***	39%
**Very severe obesity**	157.3***	39%	144.0***	36%	301.3***	75%
**45-64 (N=990831), average estimated expenditure = 867 euro**						
**Underweight**	−54.25***	−6%	180.0***	21%	125.7***	14%
**Overweight**	128.2***	15%	−45.25***	−5%	82.92***	10%
**Obesity**	257.0***	30%	−22.30***	−3%	234.7***	27%
**Severe obesity**	370.1***	43%	82.05***	9%	452.2***	52%
**Very severe obesity**	436.8***	50%	156.2***	18%	593.0***	68%
**65+ (N=780233), average estimated expenditure = 2193 euro**						
**Underweight**	−235.1***	−11%	468.4***	21%	233.4***	11%
**Overweight**	202.2***	9%	−127.3***	−6%	74.87***	3%
**Obesity**	381.9***	17%	−79.79***	−4%	302.1***	14%
**Severe obesity**	504.0***	23%	215.2***	10%	719.3***	33%
**Very severe obesity**	524.6***	24%	265.5***	12%	790.1***	36%

*** p<0.01

** p<0.05

* p<0.1, each *p value* refers to a t test for equality of means for each category with respect to normal weight category

- Average estimated expenditure for each sample was obtained as the predicted expenditure from the health expenditure equation, calculated for the normal-weight individuals, and at means of all other regressors.

- Indirect marginal effects for each BMI category were computed as the sum of nonlinear combinations of parameters estimated within each pathology-specific equation with the respective pathology-specific parameter estimated within the health expenditure equation.

- Direct marginal effects for each BMI category were obtained as relative parameter estimates from the health expenditure equation.

- Overall marginal effects for each BMI category was computed as the sum of the respective direct and indirect marginal effects.

**Figure 1:**
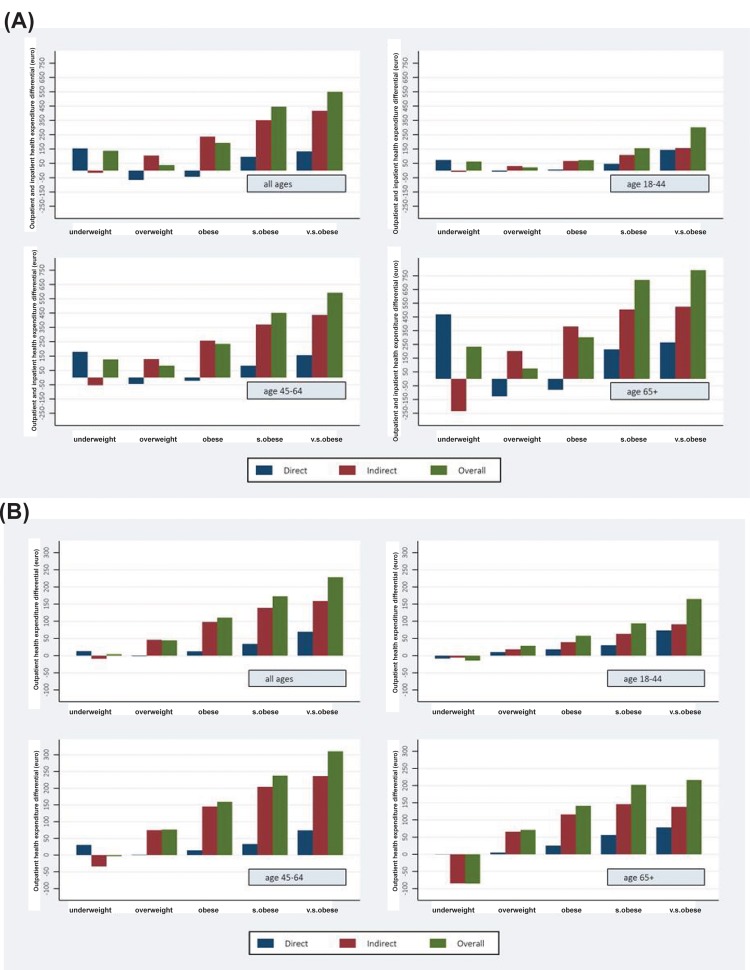
Total (a) and outpatient (b) health care expenditure Decomposition of differences in health care expenditure (direct, indirect and overall costs) by age group and BMI category compared to normal‐weight individuals (euro per year) for outpatient (**b**) and total (**a**) health expenditure. Note: - Indirect marginal effects for each BMI category were computed as the sum of nonlinear combinations of parameters estimated within each pathology‐specific equation with the respective pathology‐specific parameter estimated within the health expenditure equation. ‐ Direct marginal effects for each BMI category were obtained as relative parameter estimates from the health expenditure equation. ‐Overall marginal effects for each BMI category was computed as the sum of the respective direct and indirect marginal effects.

As shown in Figure 1, after adjusting for age, sex, geographic residence, and GPs heterogeneity, there was a J-shaped association between BMI and overall (direct and indirect) total health care expenditure, which was stronger among middle-aged and elderly individuals. Total health care expenditures among the middle-aged (45-64 yrs old) individuals with overweight, obesity, severe obesity and very severe obesity were 10%, 27%, 52% and 68% higher, respectively, than among those with BMI of 18.5 to 24.9 (p=0.0001) (third panel of Table 3). In absolute terms, outpatient costs were more strongly related to BMI among individuals aged 45 to 64 years. The annual mean costs among the overweight, obesity, severe obesity and very severe obesity patients were 76, 159, 237, and 310 euro higher, respectively, than in normo-weight individuals, which translates in a cost increase of about 18%, 38%, 57% and 75% for each BMI category, respectively (third panel of Table 2). In contrast, total costs were more strongly related to BMI among individuals aged 65+ years. The annual differential mean costs among the patients with overweight, obesity, severe obesity and very severe obesity were 75, 302, 719, and 790 euro higher, respectively, than in normal weight individuals (fourth panel of Table 3). Total overall (direct and indirect) costs were also significantly higher in underweight individuals than in normal weight individuals in all subsamples (Table 3). In particular, annual mean total costs among all underweight individuals were 138 euro higher than in the normo-weight subjects, which translates in a cost increase of 13% (first panel of Table 3).

### Indirect and direct costs

The share of indirect costs within overall outpatient costs was the largest in overweight and obese men and women aged 45-64 years (Table 2). Moreover, the indirect costs of the underweight subjects were lower than those of the normo-weight individuals for each age group, and this differential was more pronounced in the elderly, amounting to 11% (Table 2).

In terms of total outpatient and inpatient health expenditure, direct costs were negative in the overweight and obesity groups, suggesting that after correcting for BMI-related pathologies, these patients on average had lower health care expenditures than normo-weight individuals. This direct cost differential turned positive for the category with severe and very severe obesity, and was particularly pronounced in the elderly (Table 3). Moreover, the total indirect costs in the underweight individuals were significantly lower than in the normoweight subjects, and this differential was particularly high in the elderly (fourth panel of Table 3). Finally, the total direct costs in the underweight men and women were substantially higher than in the elderly normo-weight individuals.

### Relative effects of BMI and adiposity-associated comorbidities on health care costs

**Figure 2:**
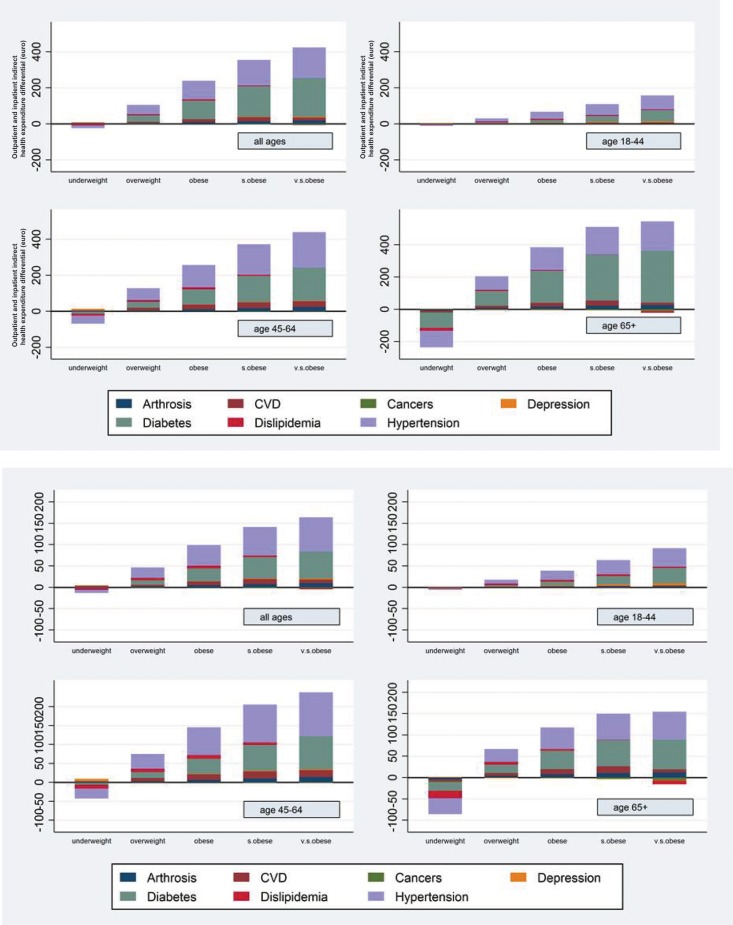
Total (a) and outpatient (b) indirect health care expenditure Decomposition of indirect health care expenditure by pathology, age group and BMI category compared to normo‐weight individuals (euro per year) for outpatient (**b**) and total (**a**) health expenditure. Note: Pathology specific indirect marginal effects for each BMI category were computed as the nonlinear combinations of parameters estimated within each pathology‐specific equation with the respective pathology‐specific parameter estimated within the health expenditure equation.

As shown in figure 1, based on SURE analysis, the indirect costs of excess body weight in individuals with overweight and obesity explain the great majority of overall BMI-related costs both in terms of outpatient and total health care expenditures. Moreover, as shown in figure 2, when hypertension, diabetes and CVD were accounted for, the age-adjusted relation between BMI and total and outpatient health care costs was to a large extent eliminated.

## DISCUSSION

In this study, we evaluated the disease burden, and the direct and indirect effects of BMI on health care costs, in a large population of 557,145 men and women for whom accurate anthropometric, clinical and medical cost data collected by general practitioners were available. First, our data show a sharp increase in the proportion of individuals affected by 2 or more age-associated chronic diseases with raising BMI. Second, our data show that after adjusting for age, sex, geographic residence, and GPs heterogeneity, BMI still has a relevant effect on both inpatient and outpatient health care expenditure. We found a strong J-shaped association between BMI and total health care costs, which was more pronounced in middle-aged and older adults. Third, our results demonstrated that hypertension, diabetes and CVD account for the largest share of outpatient and total health care expenditures.

Many studies have attempted to estimate the health care costs attributable to excess body weight. However, most published studies so far have estimated BMI-related costs by using a top-down approach which quantifies attributable fractions of costs associated with adiposity-related diseases by modelling group and individual level data collected from National Health interview surveys [5-11]. Moreover, in these studies the determinants of BMI and the prevalence of chronic diseases were mostly self-reported and, therefore, subjected to bias [31]. To our knowledge, this is the first study to determine the direct and indirect health care costs by using a prevalence-based approach that combines data from a large observational dataset, containing computer-based patient records with accurate anthropometric and clinical information and precise medical cost data collected by general practitioners. Our data are generally in line with the estimates of other studies [21, 22, 25] showing that total health care expenditure of the overweight is around 3% higher than in normal weight individuals, while patients with obesity, severe obesity and very severe obesity spend respectively 18%, 41% and 50% more than their normal-weight counterparts.

Interestingly, we found that BMI-related costs vary substantially across age groups. With respect to normoweight individuals, the highest overall (direct plus indirect) outpatient expenditure differentials in absolute terms were found in the 45-64 age group, and in the 65+ age group for total out- and in-patient health care costs. This finding suggests that outpatient health care utilization in terms of drugs, medical visits and diagnostic tests resulting from excess body weight is particularly higher in the 45-64 age group. However, when inpatient expenditure (hospitalizations) was accounted for, the 65+ age group patients generated in absolute terms the majority of the BMI related health care costs.

It is essential, however, to establish the extent to which one or more obesity-related medical conditions may account for the variation in health care costs by BMI. Our sophisticated multivariate regression SURE analysis indicated that much of the increased costs can be attributed to three very prevalent chronic diseases: hypertension, type 2 diabetes and CVD. These data are in agreement with previous estimates of other studies [32, 33]. However, this does not mean that BMI is not related to increased health care expenditure through other channels, because in our study individuals with severe and very severe obesity had high direct health care costs, net of the frequent adiposity-related medical conditions.

The prevalence of overweight and obesity in Italy, as in many other developed and developing countries, has been increasing steadily in the last few years [1, 2, 34]. Our data show that the rise in body weight is associated with a strong increase in the prevalence of several chronic diseases, including type 2 diabetes, hypertension, dyslipidemia, CVD, depression and arthrosis, especially in individuals aged 55+ years. Moreover, our data show that as BMI increases, the percentage of younger and older adults with two or more adiposity-related medical conditions increases several fold, whereas the proportion of individuals with no chronic diseases diminishes by 2 fold in the 18 to 55-year-old age group and by 3 folds in individuals over 55-years-old.

The results of our study provide firm evidence that the impact that excess body weight has on a set of chronic diseases, represents the largest component of health care expenditures. Considering our marginal BMI-related costs and the official statistics of obesity prevalence of the Italian adult population [35], we estimated that the overall BMI-related costs amount approximately to 4% of total health care expenditure of the Italian national health service (i.e. 4.5 billions of euro per year). This estimate, based on the self- and under-reported official prevalence rates of BMI among the Italian population represents a lower bound of the real costs of excessive adiposity in Italy. In fact, this estimate is slightly lower than the 5-10% found for the USA [23, 26, 29, 30], and 4.5% for the UK [28], while somewhat higher than 2.3-3.5% found for Switzerland [27].

### Strengths and limitations

It is important to highlight the strengths and limitations of this study. The use of clinical data (e.g. weights, heights, chronic disease diagnosis, test results, drug prescriptions, outpatient diagnostic tests, specialist visits and hospital admissions), collected for a large sample of patients and entered in an up-to-date computer-based database by trained GPs, is a major strength of this study. In addition, it is important to stress that the Italian National Health Service is a public and universalistic system, which provides substantially free health services for all citizens. According to OECD Health Data, in 2012 about 87% of medical services in Italy was publicly financed. This setting favours the external validity of the study and minimizes the selection problems related to presence of private insurance plans. Finally, the use of a multi-equation recursive model (SURE) to calculate the relative effects of BMI and adiposity-associated chronic diseases on health care costs represents another strength of this study, since it provides better and more efficient estimates of the cost of obesity and allows to study direct and indirect effects of BMI of health care expenditure. One major limitation of this study is the lack of alternative measures of adiposity, such as waist circumference or waist-hip ratio. Moreover, the analysis could potentially benefit by introducing covariates such as smoking or socio-economic status, enhancing the precision of the coefficient estimates, which are not available within this dataset. Finally, the estimates might suffer from a downward bias, as the control group includes individuals who, while not being affected by the diseases included in this analysis, may have had other pathologies, which may increase health expenditure with respect to the overall population.

## METHODS

### Sources of data

The present analysis was based on data of 557,145 men and women, older than 18 years, who were observed at different times between 2004 and 2010, amounting to a total of 2,705,211 observations. The anthropometric and clinical data have been extracted from the Health Search/CSD Patient Database (HS), an Italian general practice registry that includes data obtained from computer-based patient records of a selected group of 700 general practitioners (GP), homogeneously distributed across all Italian regions, covering a patient population of over a 1.8 million between 2004-2010. The GPs voluntarily agreed to collect patient information and to attend specific training courses for data entry [13]. The HS database contains patient demographic data that are linked through the use of an encrypted patient code with their medical records (diagnoses, prescribed tests results), drug prescription information (medication name, date of filled prescription, and number of days' supply), hospital admissions, and date of death. To be considered for participation in epidemiological studies, GPs should meet “up-to-standard” quality criteria pertaining to the levels of coding, prevalence of well-known diseases, mortality rates, and years of recording [13]. The HS database complies with the European Union guidelines on the use of medical data for research, and has previously been demonstrated to be a valid data source for scientific research [14-16]. GPs collected this information on daily basis. However, in this study the records have been collapsed to obtain yearly aggregates. Finally, these data have been merged, at patient level, with data from the SiSSi (Simulazione Spesa Sanitaria Italiana – Simulation of the Italian Health Care Expenditure) project, which includes information on prices and tariffs for drugs, outpatient visits, diagnostic tests and hospitalization visits. By multiplying health care service utilization data from the HS with price and tariff data from the SiSSi project we obtained detailed information on public health care expenditure at the patient level [17-19].

### Model structure

Obesity-related health care expenditure is generally studied with a cost-regression approach [20-30]. However, this method is likely to produce biased estimates, since it does not analyze the mechanisms at play between BMI and health care expenditures. Therefore, we have built a multi-equation recursive model within a Seemingly Unrelated Regression Estimator (SURE) approach to quantify: *(1)* indirect costs, resulting from the impact that BMI has on a set of chronic diseases commonly attributable to obesity, which in turn influence health expenditure; and *(2)* direct costs, defined as the residual impact of BMI independent of adiposity-associated chronic diseases, after adjusting for age, sex, geographic residence, and GPs heterogeneity. The statistical model consisted of eight equations, estimated simultaneously, of which the first 7 represent the sets of obesity-related chronic diseases *(1)* type 2 diabetes mellitus; (*2)* hypertension; (*3)* dyslipidemia; (*4)* cardiovascular diseases: coronary artery disease, ischemic stroke, congestive heart failure; (*5)* cancers: breast, colon, and prostate cancer, (*6)* arthrosis: hip and knee arthrosis; (*7)* depression, while the last equation modeled the health care expenditure as a function of the above chronic conditions plus BMI.

### Dependent variables

The empirical model comprised 8 equations, and hence there were 8 dependent variables: 7 dummy variables indicating the presence of one of the selected diseases at patient level plus the per-capita health care expenditure variable. We used two definitions of health expenditure: “outpatient” health care expenditure and “total” health care expenditure (i.e. inpatient and outpatient medical costs). Precise data on health care expenditure were obtained from a merge of Health Search CSD-LPD database with data on costs and tariffs of drugs, outpatient visits, diagnostic tests, and hospitalizations from the SiSSI Project. Furthermore, since the Health Search CSD-LPD PCP's may underreport the events of hospital admissions, we reweighted the Health Search CSD-LPD hospitalization rates with the hospital admission data collected by the Italian National Institute of Statistics (ISTAT). The weighting procedure stratifies the patient population by region, age (18-44, 45-64 and 65 and older), gender and BMI level in order to match cell groups of individuals in the Health Search CSD-LPD and ISTAT datasets. Subsequently, average hospitalization costs for the same cell groups were computed from the Hospital Discharge dataset of the Italian Ministry of Health and assigned to the Health Search CSD-LPD population.

### Covariates

BMI was calculated as weight in kilograms divided by the square of height in meters. Study participants were divided in 6 groups with: normal weight (BMI, 18.5-24.99 [the reference group]), underweight (BMI between 15 and 18.49), overweight (BMI, 25.0-29.99), obesity (BMI, 30.0-34.99), severe obesity (BMI, 35.0-39.99) or very severe obesity (BMI >40.0). Since health expenditure may differ due to GPs practice heterogeneity, we controlled for GPs behavior, including variables such as the average number of patient contacts, the average number of prevention visits, the number of overall patients registered with the GPs practice and the average unit price of the prescribed drugs. Finally, we accounted for patient heterogeneity by controlling for age, gender, and region of residence.

### Statistical analysis

In econometrics, the seemingly unrelated regression equations (SURE) model is a generalization of a linear regression model that consists of several regression equations, each having its own dependent variable and potentially different sets of exogenous explanatory variables [20]. Analytically, the structure of a SURE model employed in this analysis can be represented by the following 8 equation model:

$$P_{jit}=\alpha_{j0}+B_{j}\;BMI_{it-1}+\delta_{j}X_{it}+\theta_{j}GP_{it}+\varepsilon_{jit}$$

$$HE_{it}=\theta_{0}+\Sigma_{j=1}^{7}\lambda_{j}P_{jit}+\pi BMI_{it-1}+\rho X_{it}+\sigma GP_{it}+\eta_{it}$$

where *j = 1,..,7* represents the sets of pathologies, *i* stands for individuals, *t =2004,...,2010* for years, and where *P* is the set of seven pathologies' indicator dummy variables, *HE* denotes health expenditure in euro per year, *BMI* is the vector of body weight dummy variables categories observed in the year preceding the diagnosis of pathologies and health expenditure realization; X is a set of control variables including age, gender, region of residence and year time dummies; and GP is a vector of variables including physician characteristics. Finally, εjit and ηit are idiosincratic error terms. The assumption of the model is that error terms are independent across time, but may have cross-equation contemporaneous correlations. Thus we assume that E[*ε_ir_ ε_is_* | *X*] = 0 whenever *r ≠ s*, whereas E[*ε_ir_ ε_jr_* | *X*] = σ_*ij*_. Although Ordinary Least Squares (OLS) estimate of our model parameters are consistent, generally they are not as efficient as the SURE method, which uses the feasible generalized least squares (FGLS) method with a specific form of the variance-covariance matrix. The analysis was conducted separately for the whole sample of patients as well as for subsamples by age classes (18-44 year olds, 45-64 year olds and 65+ year olds). The coefficient estimates were expressed as marginal effects. For the full sample, the reference category was a male, resident of the Piedmont region in North Italy, in the normal weight BMI category, not affected by any of the pathologies considered in the study, observed for the first time in 2004 and aged between 18 and 44. This last condition was not considered when dealing with the subsamples by ages. The parameter estimates described the impact of each covariate on individual health care expenditure. The statistical analysis was performed using Stata software version 13 (Stata Corps).

## CONCLUSION

Based on one of the largest datasets used in this literature, the results of this study reinforce the concept that overweight and obesity increases the risk of developing multiple and costly chronic diseases. Our findings demonstrate the adverse impact of increased BMI on outpatient and total healthcare costs, especially in middle-aged and elderly individuals. They also show that hypertension, type 2 diabetes and CVD are responsible for a large part of these BMI-related health care expenditures. The knowledge of these costs will be useful for future economic analysis of preventive and treatment interventions, such as long-term, comprehensive national initiatives that tackle the basic causes of poor diet quality and sedentary lifestyles.
